# Laparoscopic surgery to treat leiomyosarcomas of the sigmoid colon:a case report and literature review

**DOI:** 10.1186/s40792-019-0579-8

**Published:** 2019-02-12

**Authors:** Masashi Yahagi, Yoshiyuki Ishii, Atsuko Hara, Masahiko Watanabe

**Affiliations:** 1 Department of Surgery, Kitasato University Kitasato Institute Hospital, 5-9-1 Shirokane, Minato-ku, Tokyo, Japan; 2 Department of Pathology, Kitasato University Kitasato Institute Hospital, 5-9-1 Shirokane, Minato-ku, Tokyo, Japan; 3 Department of Surgery, Kitasato University School of Medicine, 1-15-1 Kitasato, Sagamihara, Kanagawa Japan

**Keywords:** Leiomyosarcoma, Colon, Laparoscopic surgery

## Abstract

**Background:**

Leiomyosarcomas (LMSs) of the colon are extremely rare and highly aggressive. Although treatment of gastrointestinal LMS is not standardized, surgical resection is generally performed. The fact that the tumors are usually large at the time of diagnosis may explain why no report on laparoscopic resection of a colonic LMS has appeared.

**Case presentation:**

A 46-year-old male presented with hematochezia 1 month in duration. Abdominal examination including palpation was normal. The levels of several blood tumor markers were normal. Colonoscopy revealed a polypoid lesion approximately 30 mm in diameter in the sigmoid colon 30 cm from the anal verge. Contrast-enhanced computed tomography revealed that the tumor was 28 mm in diameter, and that no lymph node or distant metastasis was apparent. Histopathological examination of a biopsy specimen revealed spindle-shaped cells exhibiting significant nuclear atypia and a trabecular proliferation pattern upon hematoxylin-eosin staining. Immunohistochemically, the sample was positive for SMA and desmin, and negative for c-kit, DOG-1, CD34, and S-100. Furthermore, the Ki-67 index was > 50%. We thus diagnosed a leiomyosarcoma of the sigmoid colon without any metastasis. We performed laparoscopic sigmoid colectomy and regional lymphadenectomy using five trocars. After complete curative resection, a colorectal end-to-end anastomosis was created employing the double-stapling technique. All surgical margins were negative, and no lymph node metastasis was observed. The postoperative course was uneventful, and the patient was discharged 9 days after operation. No recurrence was noted to 1 year after surgery.

**Conclusions:**

We report the first case of a colonic LMS treated via laparoscopic surgery. Although further work is necessary to assess prognosis and to develop the treatment further, laparoscopic surgery to treat small colonic LMSs may be feasible, being both minimally invasive and curative.

## Background

Leiomyosarcoma (LMS) of the colon is an extremely rare and highly aggressive neoplasm [1, 2]. After it was found that c-kit gain-of-function played an important oncological role in gastrointestinal stromal tumors (GISTs), many tumors previously diagnosed as LMSs turned out to be GISTs [3]. Today, true LMS is immunohistochemically distinguished from other mesenchymal tumors by virtue of the expression of smooth muscle actin (SMA) and desmin, but not GIST markers (KIT, CD34, and DOG1) or the schwannoma marker (S100) [4]. As an LMS of the colon is less symptomatic than an LMS of the rectum, colonic tumors are often large [2], and no instance of laparoscopic resection has yet been reported. Here, we present the first case of LMS of the sigmoid colon that was safely and curatively resected via laparoscopic surgery.

## Case presentation

**Fig. 1:**
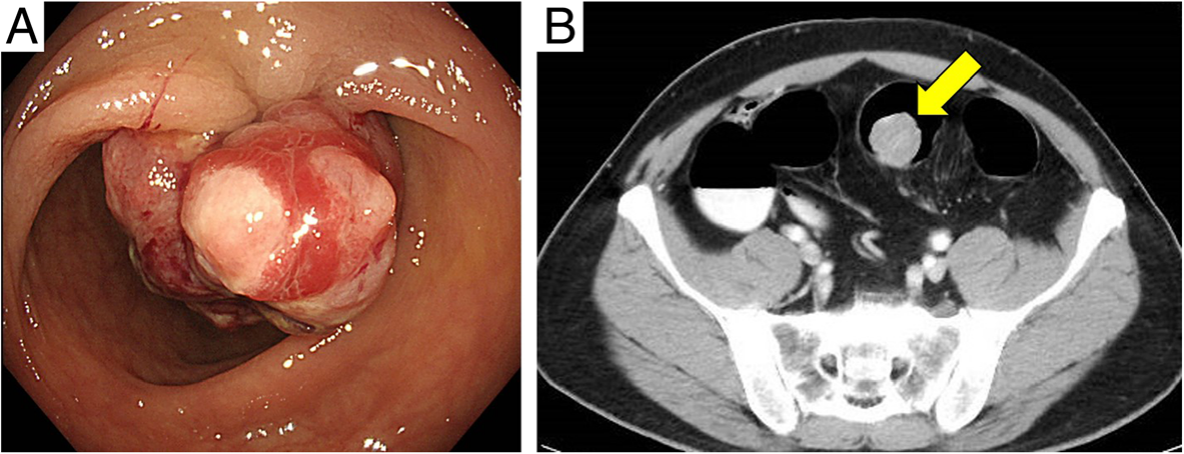
**a** Endoscopic findings: colonoscopy revealed a large polypoidal tumor in the sigmoid colon. **b** Contrast-enhanced computed tomography findings: the position of the tumor is indicated; no metastasis to lymph nodes or a distant site was evident

**Fig. 2:**
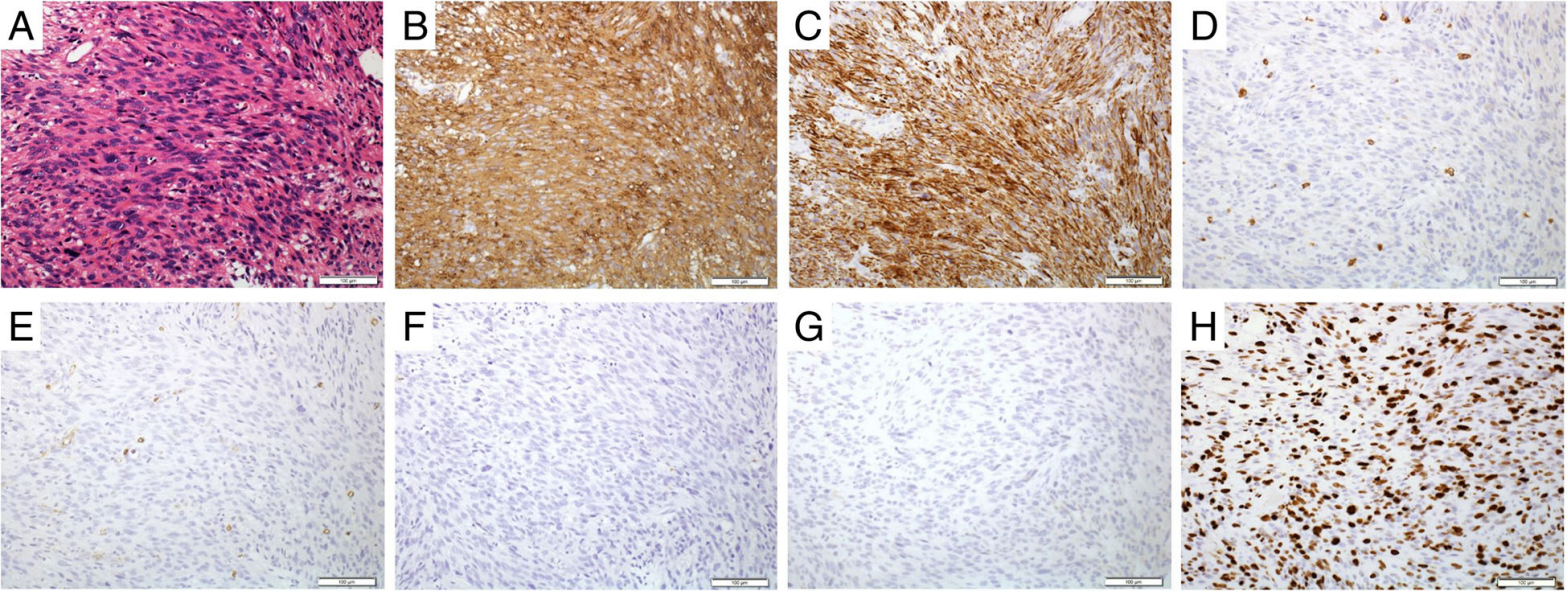
Microscopic and immunohistochemical findings. **a** Hematoxylin-eosin staining revealed spindle-shaped cells exhibiting significant nuclear atypia. The tumor was immunohistochemically positive for α-SMA (**b**), desmin (**c**), and Ki-67 (**h**), but negative for c-kit (**d**), CD34 (**e**), S-100 (**f**), and DOG-1 (**g**) (× 20)

A 46-year-old male presented to our institution complaining of hematochezia 1 month in duration. His past medical history included coronary vasospastic angina and diabetes mellitus that was treated with insulin. He drank socially and had a 50-pack-year smoking history, but had quit smoking 4 months prior. Abdominal examination including palpation was normal. The levels of carcinoembryonic antigen (4.2 ng/mL) and carbohydrate antigen 19–9 (13.2 U/mL) (commonly measured blood tumor markers) were normal. Colonoscopy revealed a polypoid lesion approximately 30 mm in diameter in the sigmoid colon 30 cm from the anal verge (Fig. 1a). Histopathological examination of a biopsy specimen revealed spindle-shaped cells exhibiting significant nuclear atypia and a trabecular proliferation pattern on hematoxylin-eosin staining. Immunohistochemically, the tissue was positive for SMA and desmin and negative for c-kit, DOG-1, CD34, and S-100. Furthermore, the Ki-67 index was > 50% (Fig. 2). Contrast-enhanced computed tomography from the chest to the pelvis revealed a tumor 28 mm in diameter in the sigmoid colon and the absence of involved lymph nodes and any distant metastasis (Fig. 1b).

**Fig. 3:**
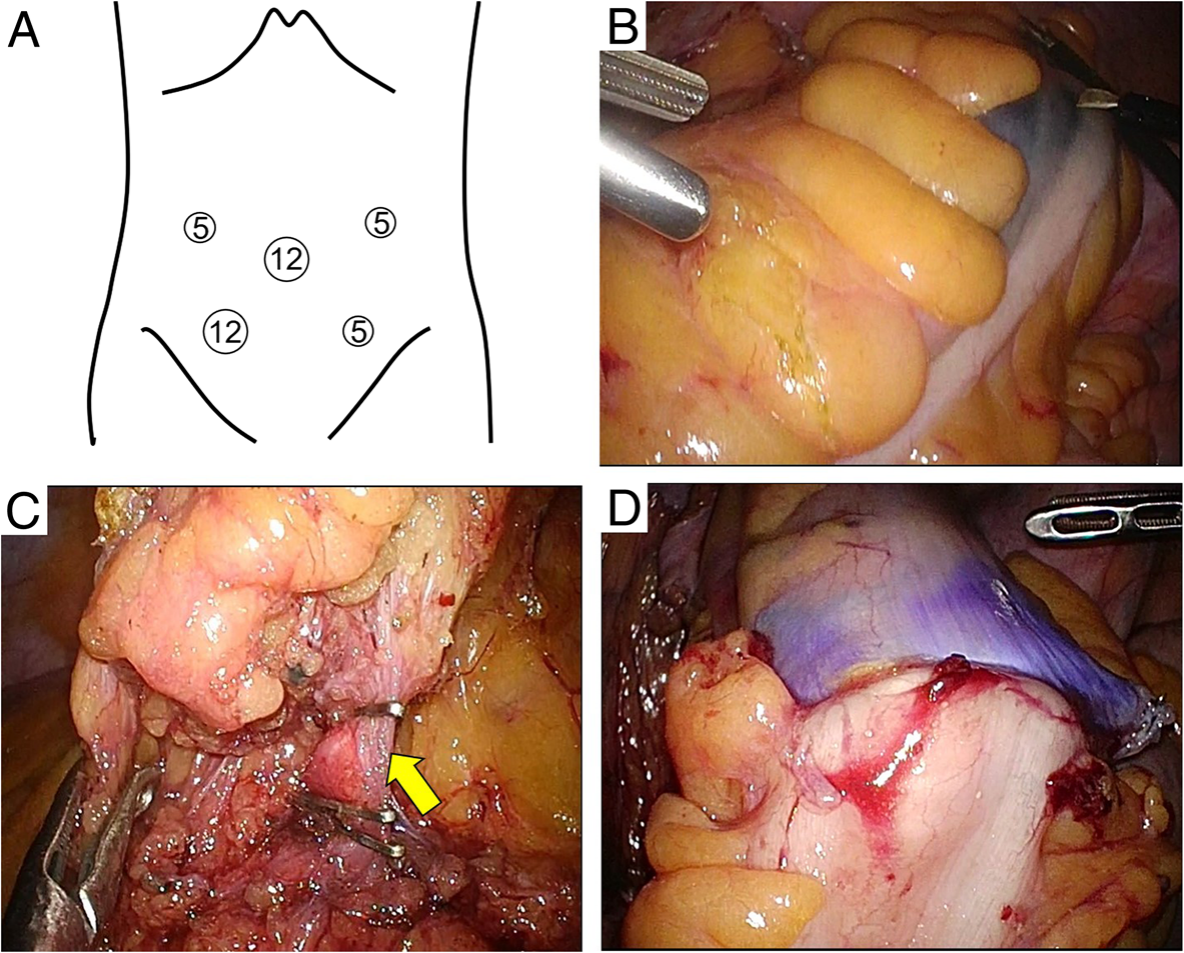
The sites of the portals and intraoperative views. **a** Portal sites: the first trocar was a 12-mm umbilical trocar and another such trocar was placed in the right lower abdomen, followed by three 5-mm trocars in the left lower and both sides of the abdomen. **b** The tattoo indicates the tumor site. **c** The arrow indicates the central ligation point of the inferior mesenteric artery. **d** Anastomosis was performed using the double-stapling technique

**Fig. 4:**
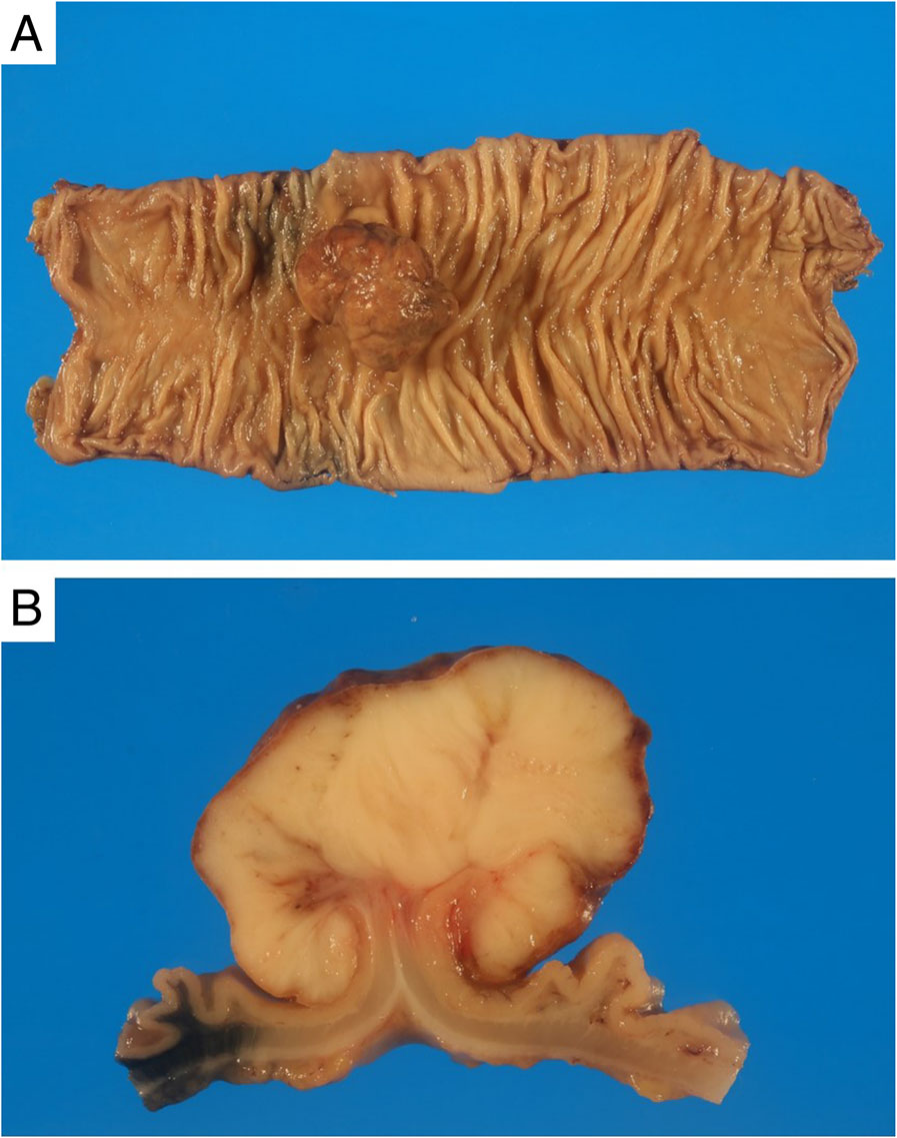
The resected specimen. **a** The tumor dimensions were 42 × 37 × 28 mm and the surface was elastic and hard. **b** The cross-section was white with a 5-mm peduncle

We diagnosed an LMS of the sigmoid colon without metastasis. We performed laparoscopic sigmoid colectomy and regional lymphadenectomy, following the concept of complete mesocolic excision and high-level central vascular ligation with curative intent for colon cancer patients. Laparoscopic surgery was performed with the aid of five trocars. The first trocar (12 mm in length) was placed in the umbilicus using an open method. Another 12-mm trocar was placed in the right lower abdomen, and three 5-mm trocars were placed in the left lower and either side of the abdomen (Fig. 3a). We identified the tattoo injected near the tumor before surgery (Fig. 3b). We dissected and mobilized the sigmoid colon by the medial-to-lateral approach in Toldt’s space and performed high-level central vascular (inferior mesenteric artery) ligation (Fig. 3c). The sigmoid colon was transected to remove a 10-cm tract margin from the tumor. After complete curative resection, a colorectal end-to-end anastomosis was created using the double-stapling technique (Fig. 3d). The tumor dimensions were 42 × 37 × 28 mm, and the surface was elastic and hard (Fig. 4a). The cross-section was white with a 5 mm peduncle (Fig. 4b). The epithelium was widely exfoliated, and the tumor per se featured trabecular proliferation of spindle cells with prominent anisonucleosis and nuclear atypia. The tumor involved the base of the muscularis propria, but was not continuous with the base. Therefore, the tumor was considered to have originated in the muscularis mucosa. All surgical margins, and lymph node vascular invasion status, were negative. Forty-four lymph nodes were harvested; no lymph node metastasis was detected. The tumor was of histological grade 2 by reference to the French Federation Nationale des Centers de Lutte Contre le Cancer (FNCLCC) system [5]; of stage IIA of the TNM classification of the 7th Edition of the American Joint Committee on Cancer (AJCC) system [6], the 7th Edition of the Union for International Cancer Control (UICC) system, and a previously published surgical staging system [7].

**Fig. 5:**
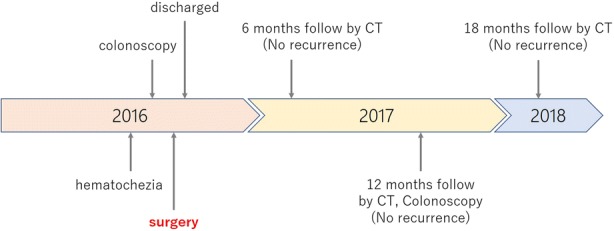
Timeline of clinical course: CT computed tomography

The postoperative course was uneventful, and the patient was discharged 9 days after the operation. He was followed-up every 6 months by contrast-enhanced computed tomography and every 12 months by colonoscopy; he did not receive adjuvant chemotherapy. There was no evidence of recurrence to 1.5 years after surgery. The clinical course of this patient is shown in Fig. 5.

LMS of the colon is often diagnosed later than LMS of the rectum. Therefore, only a few such patients can undergo laparoscopic surgery. We here report the first case of curative laparoscopic surgery to treat LMS of the colon.

LMSs originate from smooth muscle, which is widespread in the body; LMSs are most common in the retroperitoneum (including the pelvis), large blood vessels (principally the inferior vena cava), and the lower extremities [8]. Gastrointestinal LMSs of the colon are very uncommon, accounting for < 0.1% of all colorectal malignancies. Theoretically, LMSs can develop from both the muscularis propria and the muscularis mucosae; however, few reports on LMS origins have appeared. Although the muscularis propria contains more smooth muscle than the muscularis mucosae, a macroscopic polypoid LMS protruding inside the colon has often been reported [2]. Therefore, development of LMS from the muscularis mucosae (as in our case) may not be uncommon.

We searched the PubMed database using the MeSH terms “leiomyosarcoma” and “colon.” English-language reports published after 1998 investigating cases that were clearly immunohistochemically diagnosed as LMSs were selected. All cited references were also reviewed. The search yielded 197 publications, of which 26 were ultimately evaluated [9–23]. Table 1 summarizes these data and our case. The patients included 16 males and 11 females of median age 57.5 years [interquartile range (IQR), 53–65.5 years]. Tumors were in the right colon in 14 cases, and the left colon in 13, most frequently in the sigmoid colon (9 cases). The median tumor diameter was 7 cm (IQR, 3.7–10.5 cm).

**Table 1:** Summary of all selected cases and our case

Case	Reference (year)	Age	Sex	Site	Diameter (cm)	Gross appearance	Mitoses /10 HPFs	Immunohistochemistry	Procedure	Local recurrence	Metastasis	Outcome	F/U period (months)
1	[9] (2000)	54	M	D	3.2	Polypoid	> 20	α-SMA+, desmin+, c-kit−, CD34−, S-100−	OP	N/A	N/A	Death	37
2	[9] (2000)	61	M	A	4.2	Sessile	> 20	α-SMA+, c-kit-, CD34−, S-100−	OP	–	–	Survived	141
3	[9] (2000)	75	M	A	6.5	Plaque	> 20	α-SMA+, c-kit−, CD34−, S-100−	OP	N/A	N/A	Death	6
4	[9] (2000)	76	F	C	7.8	Multinodular	> 20	α-SMA+, c-kit−, CD34−, S-100−	OP	N/A	N/A	Death	7
5	[9] (2000)	36	F	S	6.5	Polypoid	> 20	α-SMA+, desmin+, c-kit−, CD34−, S-100−	OP	–	Lung	Death	38
6	[9] (2000)	66	M	A	N/A	Polypoid	8	α-SMA+, c-kit−, CD34−, S-100−	OP	–	Liver	Death	19
7	[9] (2000)	41	M	C	7.5	Pedunculated	> 20	α-SMA+, desmin+, c-kit−, CD34−, S-100−	OP	–	Humerus	Survived	185
8	[10] (2004)	65	M	D	10	Polypoid	2	α-SMA+, desmin−, c-kit−, CD34−, S-100−	OP	–	+	Death	28
9	[11] (2004)	67	F	T	5.7	Polypoid	N/A	α-SMA+, c-kit−, CD34−, S-100−	OP	–	–	Survived	12
10	[12] (2007)	77	F	S	N/A	Intramural	10–30	α-SMA+, desmin+, c-kit−, CD34−, S-100−	N/A	+	–	N/A	N/A
11	[12] (2007)	52	M	S	N/A	Intramural	10–30	α-SMA+, desmin+, c-kit−, CD34−, S-100−	N/A	–	Liver	N/A	N/A
12	[13] (2011)	70	F	S	3.7	Intramural	20	α-SMA+, desmin+, c-kit−, CD34−, S-100−	Open-Harrtman	–	–	Death	4
13	[13] (2011)	56	M	C	N/A	N/A	N/A	α-SMA+, desmin+, c-kit−, CD34−	Open-RHC	–	Liver	Survived	68
14	[14] (2012)	66	F	S	3	Intramural	20	α-SMA+, desmin+, c-kit−, CD34−, S-100−	OP	–	Liver	Death	58
15	[15] (2013)	94	F	D	25	Type 2	30	α-SMA+, c-kit−, CD34−, S-100−	N/A	–	Liver	Death	7
16	[15] (2013)	56	M	S	1	Intramural	18	α-SMA+, c-kit−, CD34−, S-100−	N/A	–	LN	Survived	60
17	[15] (2013)	78	F	S	8.5	Type 2	31	α-SMA+, c-kit−, CD34−, S-100−	N/A	–	Lung	Death	16
18	[15] (2013)	87	M	T	11	Intramural	102	α-SMA+, c-kit−, CD34−, S-100−	N/A	–	–	Death	2
19	[16] (2014)	66	F	T	4	Polypoid	> 5	α-SMA+, c-kit−, CD34−, S-100−	Open-RHC	–	–	Survived	33
20	[17] (2014)	65	M	S	N/A	N/A	N/A	α-SMA+, c-kit−	OP	–	–	Survived	12
21	[18] (2015)	46	M	T	11.8	Type 2	61	α-SMA+, c-kit−, CD34−, S-100−	Open-RHC	+	–	Survived	30
22	[19] (2015)	89	F	A	4.5	N/A	N/A	α-SMA+, c-kit−	Open-RHC	–	Liver	N/A	N/A
23	[20] (2015)	54	M	A	13	Intramural	10–12	α-SMA+, desmin+, c-kit−, CD34−	OP	+	–	Survived	> 6
24	[21] (2015)	59	M	A	10	Exophytic	< 0.5	α-SMA+, desmin+, c-kit−, CD34−, s-100−	Open-RHC	–	–	Survived	8
25	[22] (2016)	82	M	C	2.2	Polypoid	20	desmin+, c-kit−	Open-RHC	–	–	Survived	14
26	[23] (2016)	51	F	D	4	Type 2	10	α-SMA+, desmin+, c-kit-, CD34-, s-100-	OP	–	–	Survived	31
27	Our case	46	M	S	4.2	Polypoid	39	α-SMA+, desmin+, c-kit-, CD34-, S-100-	Lap-sigmoidectomy	–	–	Survived	17

*M* male, *F* female, *C* cecum, *A* ascending colon, *T* transverse colon, *D* descending colon, *S* sigmoid colon, *N/A* not available, *SMA* smooth muscle actin, *OP* open partial colectomy, *RHC* right hemicolectomy, *Lap* laparoscopic, *LN* lymph node, *F/U* follow-up

Surgery is the standard treatment for localized soft tissue and visceral sarcomas [24]. In all cases, surgery was performed, but no standard therapeutic strategy for gastrointestinal LMSs has yet been established. Lymph node metastasis of gastrointestinal LMSs is rather uncommon; however, lymph node dissection is advisable if this is not excessively invasive, because lymph node metastasis has been reported even in those with small poorly proliferating tumors [one such tumor was 1 cm in diameter and exhibited 18 mitoses/50 high-power fields (HPFs)] [15]. The standard chemotherapies for advanced soft tissue and visceral sarcomas are first-line anthracyclines, with doxorubicin-plus-dacarbazine as an alternative [24]. Presently, chemotherapy plays a limited role in LMS treatment. Factors prognostic of LMS are unclear because LMSs are rare, but tumor diameter ≥ 5 cm has been reported to be poorly prognostic [15]. Therefore, minimally invasive laparoscopic surgery should be performed only when LMSs are < 5 cm in diameter, as in our case.

## Conclusion

We report an LMS of the colon treated via laparoscopic surgery. Although further work is necessary to assess prognosis and to develop the treatment further, laparoscopic surgery to treat small colonic LMSs is feasible, minimally invasive, and curative.

## Abbreviations

AJCC: American Joint Committee on Cancer
FNCLCC: French Federation Nationale des Centers de Lutte Contre le Cancer
GIST: Gastrointestinal stromal tumor
HPF: High-power field
IQR: Interquartile range
LMS: Leiomyosarcoma
SMA: Smooth muscle actin
UICC: Union for International Cancer Control
